# Ureteral calculi in octogenarians and nonagenarians: Contemporary in-hospital management—A joint study by the endourological section of the Austrian Association of Urology

**DOI:** 10.1371/journal.pone.0280140

**Published:** 2023-01-17

**Authors:** Martin Drerup, Mehmet Özsoy, Clemens Wehrberger, Matthias Lenz, Christian Ramesmayer, Philipp Stolzlechner, Johannes Zanier, C. E. Falkensammer, Ivan Handjev, Andreas Wasserscheid, Stephan Seklehner

**Affiliations:** 1 Department of Urology, Paracelsus Medizinische Privatuniversität Salzburg, Salzburg, Austria; 2 Department of Urology, Krankenhaus der Barmherzigen Brüder Salzburg, Salzburg, Austria; 3 UROMED KompetenzZentrum Urologie, Vienna, Austria; 4 Department of Urology, Klinik Donaustadt, Vienna, Austria; 5 Department of Urology, Universitätsklinikum Krems an der Donau, Krems an der Donau, Austria; 6 Department of Urology, Tauernklinikum Zell am See, Zell am See, Austria; 7 Department of Urology, Medizinische Universität Innsbruck, Innsbruck, Austria; 8 Department of Urology, Klinik Favoriten, Vienna, Austria; 9 Department of Urology, Klinikum Klagenfurt am Wörthersee, Klagenfurt am Wörthersee, Austria; 10 Department of Urology, Landesklinikum Baden-Mödling, Baden bei Wien, Austria; Stanford University School of Medicine, UNITED STATES

## Abstract

**Aim:**

To assess contemporary in-hospital management of octogenarians and nonagenarians with ureteral calculi.

**Materials and methods:**

Review of patients aged ≥80 years hospitalized due to ureteral calculi. Data was extracted from eight Austrian centers of urology. Stone and patient related data were recorded. Treatment patterns in acute and elective settings were assessed.

**Results:**

A total of 759 patients hospitalized with ureteral calculi were analyzed. Out of them, 643 were octogenarians (80-89years) and 116 nonagenarians (90–99 years). In an acute setting, simple de-obstruction with urinary diversions outnumbered active stone treatments like URS and SWL (62.6% vs. 26.9% vs. 10.5%). Decision making whether patients underwent active stone treatment was driven by stone location (OR = 0.28, p<0.0001), impaired renal function (OR = 0.28, p = 0.01) and indwelling urethral catheters (OR = 0.23, p = 0.01) but not by age or extend of mobility (all p>0.05). In elective settings, 81.5% of procedures were active stone treatments–mainly URS (76.9%), while DJ stent or nephrostomy replacements were noted in 14.2% and 4.3%. Octogenarians (OR = 14, p<0.0001) and patients capable of walking (OR = 4.51, p = 0.01) had significantly higher odds of receiving active stone. Stone free rates and complications rates with URS were similar between octogenarians and nonagenarians (p = 0.98 and p = 0.58).

**Conclusion:**

In acute settings, age and extend of mobility were not found to be independent predictors for active stone treatment. In elective settings, after having received urinary diversions, reduced mobility and nonagenarians were less likely to undergo stone removal treatments. Safety and efficacy of URS seems to be similar in octogenarians and nonagenarians.

## Introduction

Urolithiasis is a common disease with a prevalence between 5% and 12% dependent on geographic region [1]. Stone prevalence has increased in the last decade and is expected to rise further [2, 3]. Patients above the age of 65 encompass 10–15% of stone population already. The reasons are well known and diverse. There is evidence that obesity, diabetes mellitus, hypertension, chronic kidney disease, and cardiovascular problems are drivers for stone disease. Age is frequently associated with such conditions [2, 4]. Due to demographic changes, the global population is aging. Today, more than 125 million people worldwide can be regarded as very old as they are above 80 years of age. By 2050, it is expected that 434 million people will be older than 80 years [5, 6].

As consequence, an increasing number of very old will need to be treated in the future [7]. Although age is not a disease itself, elderly may have a higher risk of comorbidities and a poorer performance status. This may lead to higher treatment related morbidity and mortality compared to younger patients [1, 8].

Guidelines such as the one provided by the European Association of Urology support clinicians with treatment algorithms in stone patients [9]. However, very old patients are currently not explicitly addressed in the guidelines as the so far published literature on elderly patients is scarce [5, 9–15]. Treatment decisions in very old patients and those who have a reduced mobility are, mostly, driven by clinicians’ experience, geriatric assessment and patient’s performance status. In an acute setting due to ureteral stones, de-obstruction with or without active stone treatment are well accepted treatment modalities. In case of the geriatric patient with functional impairments clinicians must rely on their clinical experience whether they simply de-obstruct or if they aim for active stone treatment.

The present study was conducted in a multi-institutional setting in order to better understand the contemporary management of ureteral stones in patients above 80 years of age.

## Materials and methods

After having received institutional review board approvals from participating study sites, records of all hospitalized patients ≥80 years due to ureteral calculi were reviewed in a retrospective fashion.

Clinicians from eight Austrian centers (Landesklinikum Baden-Mödling, Klinik Donaustadt, Universitätsklinik Innsbruck, Landesklinikum Zell am See, Klinik Favoriten, Universitätsklinikum Krems, Landesklinik Salzburg, Landesklinik Klagenfurt) reviewed their patient records between 1.1.2000 and 31.12.2019. No particular exclusion criteria existed. The study is a joint project of the endourological section of the Austrian Association of Urology *(Arbeitskreis für Endourologie und Steinerkrankungen der Österreichischen Gesellschaft für Urologie)*.

Patients’ age, gender, comorbidities and stone related data were extracted. Stone size was measured as the greatest diameter on native CT scan through bone window, stone location was categorized as proximal and distal with respect to the level of ureteral iliac crossing. The extent of mobility was categorized into: ‘no aid needed, walking aid like sticks, wheelchair-bound or bedridden’. As some degree of impaired mobility is likely in very old cohorts, patients capable of walking (‘no aid needed’ and ‘walking aid like sticks’) were compared to those being less mobile (‘wheelchair-bound’ and ‘bedridden’) for some statistical analyses. Additionally, American Society of Anesthesiologists (ASA) Scores were utilized to assess patient’s fitness undergoing surgery [16]. Stone free rates with URS and its complications were assessed intra-operatively by the surgeons. Complications were recorded according to Clavien-Dindo classification [17].

We distinguished between hospitalization due to an acute setting and due to an elective setting. Reason for hospitalization in acute situations as well as spontaneous stone passage rates and surgical interventions were recorded. Elective admissions and ureteral stone management after initial de-obstruction with DJ stents or nephrostomy tubes were also analyzed.

Additionally, we differentiated between octogenarians (patients aged 80–89) and nonagenarians (aged 90–99) in order to better understand treatment differences and decisions in this very old cohort. Statistical analyses, including the chi-square test, and the logistic regression analyses, were performed with SPSS, version 21 (IBM Corp, Armonk, NY). Multivariate logistic regression analyses were carried out with significant findings from univariate analyses in order to obtain independent prognostic factors influencing treatment decisions. A P value of **<**0.05 was considered to be statistically significant.

## Results

A total of 759 patients hospitalized due to ureteral stones were observed in the studied period. Out of those, 453 were hospitalized in an acute compared to 306 in an elective setting. Patients were more frequently male (54,9%) with a mean age of 85.1 years. Mean stone size was 7,4mm with slightly more stones being located proximally than distally. Nearly half of the cohort, 47.2% required physical aids or were non-mobile and suffered from several comorbidities as displayed in S1 Table.

### Acute setting

Reasons for hospitalizations in an acute setting were pain, urinary tract infections and impaired renal function (S1 Table).

The majority of patients underwent invasive therapy in an acute setting whereas conservative in-hospital management was less frequently (86.3% vs. 13.7%), with spontaneous stone passages during hospitalization being noted in 10%.

De-obstruction (primarily with ureteral stents) was the main treatment choice (62.6%), followed by active stone treatment via URS (26.9%) or *in-situ* SWL (10.5%) (S2 Table).

A breakdown of patients’ management in an acute setting is shown in S3 Table. Primary de-obstruction compared to active stone treatment was significantly more frequently done in women, bigger and proximally located stones, infection, impaired renal function, in higher ASA-scores, patients with reduced mobility, patients with indwelling urethral catheters and those taking anticoagulants (all p<0.05).

On multivariate logistic regression analyses patients with impaired renal function (OR = 0.28, p = 0.001), with indwelling catheters (OR = 0.26, p = 0.03) and proximal stone location (OR = 0.28, p<0.0001) were less likely to undergo primary active stone treatment with URS or *in-situ* SWL (S4 Table).

### Elective setting

In contrast to admissions in an acute setting, patients hospitalized in an elective setting mainly underwent active stone treatment (81.5%, See S2 Table). URS was the treatment of choice in most patients (76.9%), whereas SWL was rarely done (4.6%). Regular Ureteral stent or nephrostomy tube replacements instead of active stone treatment were noted in 14.2% and 4.3% of admissions, respectively.

Active stone treatment was significantly more frequently done in men, patients younger than 90 years, patients capable of walking and patients having less comorbidities, as well as in smaller and distally located stones (S5 Table, all p<0.05).

In regression analyses, being mobile or needing just walking aids (OR = 4.51, p = 0.01), stones sized less than 5mm (OR = 9.97, p = 0.003), history of stroke (OR = 7.39, p = 0.02) and being younger than 90 years (OR = 14, p<0.0001) were independent prognostic factors for undergoing active stone treatment instead of ureteral stent or nephrostomy tube replacements (See S6 Table).

#### Subgroup analyses: Octogenarians vs. nonagenarians, safety and efficacy of URS

Out of the 759 patients, 643 (84.7%) were octogenarians and 116 (15.3%) were nonagenarians. Nonagenarians were significantly more often dependent on walking aids, wheelchair or even bedridden and had higher ASA-scores (both p*<0*.*0001)*. Management in an acute setting did not differ significantly between the groups (p = 0.11) but in elective settings (p<0.0001) (S7 Table) Octogenarians underwent active stone treatment with URS more frequently (85.7% vs. 38.3%), whereas ureteral stent changes without URS or SWL were more commonly performed in nonagenarians (57.9% vs. 9.3%, respectively, p<0.0001). Octogenarians had a 14 fold higher chance of undergoing URS (OR = 14.02, p<0.0001, see S6 Table).

Immediate ureteroscopic stone-free rates were similar between octogenarians and nonagenarians (79.8% vs. 80%, p = 0.98). No relevant intraoperative complications were noted in 90.2% vs. 93.4% (p = 0.58).

## Discussion

In the present study we report on the outcome of very old and patients with ureteral calculi. To our best knowledge, literature addressing octogenarians or even nonagenarians with ureteral calculi is scarce [5, 18].

In our study, management of very old patients in an acute setting was not influenced by age or the extend of mobility. Both were not found to be independent predictors for treatment decision whether patients were scheduled for de-obstruction or for active stone removal.

Contrarily, patients with proximal stones, impaired renal function and pre-existing indwelling urethral catheters were less likely to undergo active stone removal via URS or *in-situ* SWL in an acute setting. The influence of urethral catheters on decision making is likely to be driven by the fact that indwelling catheters are known to be associated with an increased risk of urinary tract infections [19, 20]. Therefore, our data suggests, that treatment decisions in very old patients in acute settings does not necessarily differ from younger patients.

In elective settings after primary de-obstruction with ureteral stents or a nephrostomy tubes, in-hospital treatments in very old patients were mainly URS, whereas SWL was rarely done. However, this does not mean necessarily that SWL is underused in very old patients. SWL-procedures were not captured in our in-hospital records as they are frequently performed as outpatient procedures in our healthcare system. This might have caused a bias in our results.

Interestingly, 18.5% of all procedures in elective settings were surgical replacements of urinary diversions instead of active stone treatments. Likewise, this was driven by a low performance status of the corresponding patient with inability to undergo general anesthesia needed for URS. Stone size, reduced mobility (wheelchair-bound or bedridden) and age were identified as independent predictors for active stone treatment in elective settings.

Smaller stones were almost 10 times more likely to be actively treated than stones bigger than 1cm in patients ≥80 years (OR = 9.97, p = 0.003). Ureteroscopic stone free rates decrease with stone burden [21, 22]. Eventually this influenced treatment patterns in very old with complex stone situations with urinary diversion favoring over URS. Patients who were mobile or needed just walking aids like sticks were 4.5 times more likely to receive active stone treatment than those wheelchairs-bound or bedridden (OR = 4.51, p = 0.01).

In order to better understand treatment decisions in octogenarians vs. nonagenarians we compared those two age groups with each other. Treatment decisions did not differ significantly between them in acute but in elective settings. Interestingly, active stone treatment was significantly more frequently performed in octogenarians (OR = 14, p<0.0001). while in nonagenarians stents were more frequently replaced on a regular basis.

Some highly selected patients may need permanent urinary diversions due to their inability to undergo active stone treatment. However, one should bear in mind that ureteral stents might be associated with complications such as urinary tract infections, stent encrustations and migrations [23]. Also, quality of life may be considerably reduced due to stent related discomfort requiring medication in many subjects [24]. Therefore, active stone treatment should be favored over stent replacement if possible. Our study showed that, ureteroscopic stone free rates were similar and complication rates were comparable between octogenarians and nonagenarians.

Our data conform to the results of Yamashita et al, who analyzed the surgical outcome of stone procedures in patients with poor performance status. Evaluating a small sample of 52 patients undergoing various surgical procedures for stones (including 39 ureteroscopies in patients 76 years of age) they concluded that stone removal can be performed safely and effectively (stone free rates of 87%) [18]. This has also been concluded by other study groups albeit their patients were considerably younger than ours [5, 14, 25].

The closest to ours is the one recently published by Mager and coworkers [5]. They shared their experience of 325 patients with stone disease, who were at least 70 years of age. In contrast to our study, their patients were younger and procedures for ureteral as well as renal calculi were merged. Therefore, a broader spectrum of surgical procedures such as percutaneous nephrolitholapaxy, ureterolithothmy and pyelolithothomy was included. Comparing treatment patterns and outcomes of septuagenarians (subjects aged 70–79) with patients aged ≥80, they also noted no significantly different stone free rates between their age groups when URS was performed (p = 0.9). Also, surgical time (p = 0.4) and complication rates (p = 1.0) were similar. In their study population, 87.4% of patients underwent subsequent active-stone removing therapies after they initially received urinary diversions. The authors concluded active-stone removal to be safe and beneficial for patients aged ≥80 years.

Our study has several limitations. It was retrospective in nature. Only data from hospitalized patients could be analyzed–outpatient or office-based treatments or readmissions due to complication were not captured as records were not precise enough at some participating centers. Therefore, only intraoperative complications and immediate postoperative stone free rates were reported. We could not report on frailty in lack of a validated tool for its assessment in a retrospective fashion. Therefore, we only focused on patient’s mobility. Stone free rates were not determined by CT Scans. Treatment strategies differ in various hospitals. Nevertheless, capturing those differences in the multi-centric approach mirrors daily care in a European public healthcare system and it decreases the risk of treatment bias by single institutions.

Despite its limitations our study has also several strengths. To our best knowledge our study encompasses by far the greatest number of cases in the population 80+ with ureteral calculi. Also, it is to date the only one distinguishing between octogenarians and nonagenarians.

## Conclusions

In acute settings, age and reduced mobility were not found to be independent predictors for active stone treatment. In elective settings, after having received urinary diversions, patients with reduced mobility and nonagenarians were less likely to undergo stone removal treatments. Safety and efficacy of URS seems to be similar in octogenarians and nonagenarians.

## Supporting information

S1 Table Demographics. (DOCX)

S2 Table Management acute and elective setting. (DOCX)

S3 Table Breakdown of simple de-obstruction vs. active stone treatment in patients hospitalized in an acute setting. (DOCX)

S4 Table Logistic regression analyses: Likelihoods of receiving Dj-stent/PCN changes regularly instead of active stone treatment in acute settings. (DOCX)

S5 Table Breakdown of DJ/nephrostomy replacements vs. active stone treatment in patients hospitalized in an elective setting. (DOCX)

S6 Table Logistic regression analyses: Likelihoods of receiving Dj-stent/PCN changes regularly instead of active stone treatment in elective settings. (DOCX)

S7 Table Treatment patterns in octogenarians vs. nonagenarians. (DOCX)
