# Supplementary material for: Ureteral calculi in octogenarians and nonagenarians: Contemporary in-hospital management—A joint study by the endourological section of the Austrian Association of Urology
Source: PLoS One. 2023 Jan 17;18(1):e0280140. doi: 10.1371/journal.pone.0280140 (PMC9844889; doi:10.1371/journal.pone.0280140)
Supplement: S1 Table — (DOCX) [file pone.0280140.s001.docx]

|  | n= or mean | % or min-max (SD) |
| --- | --- | --- |
| Hospitalizations  Acute setting  Elective setting | 759  453  306 | 59.7%  40.3% |
| Gender  Male  Female | 417  342 | 54.9%  45.1% |
| Age | 85.1 | 80-99 (4.28) |
| Stone Size | 7.37 | 1-35 (4.38) |
| Stone location  Proximally the level of iliac vessels  Distally the level of iliac vessels  Unknown/not precisely recorded | 392  342  25 | 51.6%  45.1%  3.3% |
| Length of hospital stay | 5.86 | 1-50 (5.33) |
| Indwelling urethral catheter | 85/725 | 11.7% |
| Mobility  No aid needed  Walking aid  Wheelchair  Bedridden | 370/701  205/701  49/701  77/701 | 52.8%  29.2%  7%  11% |
| Anticoagulation | 352/759 | 46.4% |
| History of stroke | 109/758 | 14.4% |
| History of myocardial infarction | 62/759 | 8.2% |
| Coronary heart disease | 177/473 | 27.2% |
| Dementia | 108/735 | 14.8% |
| Custodianship | 25/758 | 3.3% |
| ASA-Score of hospitalized in an acute setting  1  2  3  4  5  6 | 24/363  149/363  133/363  55/363  2/363  0/363 | 6.6%  41%  36.6%  15.2%  0.6%  0% |
| Reasons for hospitalization (acute setting)  Colicky pain  Infection  Impaired renal function | 292/453  181/453  75/453 | 64.5%  40%  16.6% |
| Institutions  LK Baden  Klinik Donaustadt  UK Innsbruck  LK Zell am See  Klinik Favoriten  UK Krems  LK Salzburg  LK Klagenfurt | 185  51  95  71  66  59  113  119 | 24.4%  6.7%  12.5%  9.4%  8.7%  7.8%  14.9%  15.7% |

Table 1: Demographics
