# Supplementary material for: Ureteral calculi in octogenarians and nonagenarians: Contemporary in-hospital management—A joint study by the endourological section of the Austrian Association of Urology
Source: PLoS One. 2023 Jan 17;18(1):e0280140. doi: 10.1371/journal.pone.0280140 (PMC9844889; doi:10.1371/journal.pone.0280140)
Supplement: S2 Table — (DOCX) [file pone.0280140.s002.docx]

Table 2: Management acute and elective setting

|  | n= | % | *p-value** |
| --- | --- | --- | --- |
| Management acute setting  Conservative  Invasive | 62/453  391/453 | 13.7%  86.3% | *<0.0001* |
| Spontaneous stone passage during hospitalization | 45/453 | 10% |  |
| Invasive treatment acute setting  ureteral stent (DJ) exclusively  nephrostomy tube exclusively  ureteroscopy without DJ  ureteroscopy with DJ  SWL | 225/391  20/391  5/391  100/391  41/391 | 57.5%  5.1%  1.3%  25.6%  10.5% | *<0.0001* |
| Invasive treatment elective setting  DJ-stent replacement  nephrostomy replacement  ureteroscopy without DJ  ureteroscopy with DJ  SWL | 43/303  13/303  20/303  213/303  14/303 | 14.2%  4.3%  6.6%  70.3%  4.6% | *<0.0001* |

*Chi^2^-test
