# Supplementary material for: Ureteral calculi in octogenarians and nonagenarians: Contemporary in-hospital management—A joint study by the endourological section of the Austrian Association of Urology
Source: PLoS One. 2023 Jan 17;18(1):e0280140. doi: 10.1371/journal.pone.0280140 (PMC9844889; doi:10.1371/journal.pone.0280140)
Supplement: S3 Table — (DOCX) [file pone.0280140.s003.docx]

|  | Simple de-obstruction (DJ/PCN) | Active stone treatment (URS/SWL) | *p-value (chi^2^-test)* |
| --- | --- | --- | --- |
| Gender  Male  Female | 57.1% (117/205)  68.8% (128/186) | 42.9% (88/205)  31.2% (58/186) | *0.02* |
| Age  <90 years  ≥90 years | 60.9% (206/338)  73.6% (39/53) | 39.1% (132/338)  26.4% (14/53) | *0.08* |
| Stone size  ≤5mm  6-10mm  ≥11mm | 52.1% (75/144)  66.9% (115/172)  75.8% (50/66) | 47.9% (69/144)  33.1% (57/172)  24.2% (16/66) | *0.01* |
| Stone location  Proximal  Distal | 75% (165/220)  46.7% (77/165) | 25% (55/220)  53.3% (88/165) | *<0.0001* |
| Urinary tract infection  yes  no | 84.8% (84/99)  55.1% (161/292) | 15.2% (15/99)  44.9% (131/292) | *<0.0001* |
| Mobility  No aid needed  Walking aid  Wheelchair  Bedridden | 52.8% (103/195)  58.8% (57/97)  86.7% (26/30)  86.7% (39/45) | 47.2% (92/195)  41.2% (40/97)  13.3% (4/30)  13.3% (6/45) | *<0.0001* |
| Anticoagulation  yes  no | 68.5% (124/181)  57.6% (121/210) | 31.5% (57/181)  42.4% (89/210) | *0.03* |
| Impaired renal function  yes  no | 75.3% (55/73)  59.7% (190/318) | 24.7% (18/73)  40.3% (128/318) | *0.01* |
| Indwelling urethral catheter  yes  no | 87.7% (50/57)  56.9% (181/318) | 12.3% (7/57)  43.1% (137/318) | *<0.0001* |
| ASA-Scores  1  2  3  4  5 | 35% (7/20)  51.5% (70/136)  67.7% (84/124)  87.3% (48/55)  100% (2/2) | 65% (13/20)  48.5% (66/136)  32.3% (40/124)  12.7% (7/55)  0% (0/2) | *<0.0001* |
| Diabetes mellitus  Yes  No | 69.9% (72/103)  60.1% (173/288) | 30.1% (31/103)  39.3% (115/288) | *0.08* |
| History of myocardial infarction  Yes  No | 60% (18/30)  62.9% (227/361) | 40% (12/30)  37.1% (134/361) | *0.75* |
| Custodianship  Yes  No | 80% (8/10)  62.1% (236/380) | 20%(2/10)  37.9% (144/380) | *0.25* |
| History of Stroke  Yes  No | 69% (40/58)  61.4% (204/332) | 31% (18/58)  38.6% (128/332) | *0.28* |

Table 3: Breakdown of simple de-obstruction vs. active stone treatment in patients hospitalized in an acute setting.
