# Supplementary material for: Ureteral calculi in octogenarians and nonagenarians: Contemporary in-hospital management—A joint study by the endourological section of the Austrian Association of Urology
Source: PLoS One. 2023 Jan 17;18(1):e0280140. doi: 10.1371/journal.pone.0280140 (PMC9844889; doi:10.1371/journal.pone.0280140)
Supplement: S4 Table — (DOCX) [file pone.0280140.s004.docx]

|  | **Univariate** | **Multivariate** | | | |
| --- | --- | --- | --- | --- | --- |
|  | *p-value* |  | Odds Ratio | CI | *p-value* |
| Impaired renal function | ***0.01*** | yes  no | 0.28  1.00 | 1.3-0.61 | ***0.001*** |
| Indwelling urethral catheter | ***<0.0001*** | yes  no | 0.26  1.00 | 0.08-0.89 | ***0.03*** |
| Stone location | ***0.006*** | proximal  distal | 0.28  1.00 | 0.16-0.49 | **<0.0001** |
| Stone size | ***<0.0001*** | ≤5mm  6-10mm  ≥11mm | 0.99  0.72  1.00 | 0.44-2.25  0.33-1.57 | 0.99  0.41 |
| Urinary tract infection | ***<0.0001*** | yes  no | 0.48  1.00 | 0.23-1.03 | *0.059* |
| ASA Groups | ***<0.0001*** | 1-2  3-5 | 1.55  1.00 | 0.85-2.81 | *0.15* |
| Mobility | ***<0.0001*** | No aid/Walking aid Wheelchair/Bedridden | 1.9  1.00 | 0.72-4.99 | *0.2* |
| Anticoagulation | ***0.03*** | yes  no | 0.73  1.00 | 0.42-1-26 | *0.27* |
| Gender | ***0.02*** | male  female | 1.32  1.00 | 0.76-2.27 | *0.32* |
| Age | *0.08* |  |  |  |  |
| History of stroke | *0.28* |  |  |  |  |
| Diabetes mellitus | *0.08* |  |  |  |  |
| History of myocardial infarction | *0.75* |  |  |  |  |
| Custodianship | *0.26* |  |  |  |  |

Table 4: Logistic regression analyses: Likelihoods of receiving Dj-stent/PCN changes regularly instead of active stone treatment in acute settings.
