# Supplementary material for: Ureteral calculi in octogenarians and nonagenarians: Contemporary in-hospital management—A joint study by the endourological section of the Austrian Association of Urology
Source: PLoS One. 2023 Jan 17;18(1):e0280140. doi: 10.1371/journal.pone.0280140 (PMC9844889; doi:10.1371/journal.pone.0280140)
Supplement: S5 Table — (DOCX) [file pone.0280140.s005.docx]

|  | Change of DJ/PCN | Active stone treatment (URS/SWL) | *p-value (chi^2^-test)* |
| --- | --- | --- | --- |
| Gender  Male  Female | 12% (20/166)  26.3% (36/137) | 88% (146/166)  73.7% (101/137) | *0.001* |
| Age  <90 years  ≥90 years | 9.3% (23/246)  57.9% (33/57) | 90.7% (223/246)  42.1% (24/57) | *<0.0001* |
| Stone size  ≤5mm  6-10mm  ≥11mm | 4% (4/100)  25.9% (37/143)  29.2% (14/48) | 96% (96/100)  74.1% (106/143)  70.8% (34/48) | *<0.0001* |
| Stone location  Proximal  Distal | 25.5% (40/157)  10.2% (13/127) | 74.5% (117/157)  89.9% (114/127) | *0.001* |
| Mobility  No aid needed  Walking aid  Wheelchair  Bedridden | 0% (0/11)  12.1% (11/91)  15.4% (24/156)  66.7% (8/12) | 100% (11/11)  87.9% (80/91)  84.6% (132/156)  33.3% (4/12) | *<0.0001* |
| Anticoagulation  yes  no | 18.1% (26/144)  18.9% (30/159) | 81.9% (118/144)  81.1% (129/159) | *0.86* |
| Indwelling uretral catheter  yes  no | 22.7% (5/22)  18% (48/266) | 77.3% (17/22)  82% (218/266) | *0.59* |
| ASA  1  2  3  4 | 0% (0/11)  12.1% (11/91)  15.4% (24/156)  66.7% (8/12) | 100% (11/11)  87.9% (80/91)  84.6% (132/156)  33.3% (4/12) | *<0.0001* |
| Diabetes mellitus  Yes  No | 20.7% (12/58)  18% (44/245) | 79.3% (46/58)  82% (201/245) | *0.63* |
| History of myocardial infarction  Yes  No | 10.3% (3/43)  19.3% (53/260) | 89.7% (26/29)  80.7% (221/260) | *0.24* |
| Custodianship  Yes  No | 60% (9/15)  16.3% (47/288) | 40% (6/15)  83.7% (241/288) | *<0.0001* |
| History of stroke  Yes  No | 7% (3/43)  20.4% (53/260) | 93% (40/43)  79.6% (207/260) | *0.04* |

Table 5: Breakdown of DJ/nephrostomy replacements vs. active stone treatment in patients hospitalized in an elective setting
