# Supplementary material for: Ureteral calculi in octogenarians and nonagenarians: Contemporary in-hospital management—A joint study by the endourological section of the Austrian Association of Urology
Source: PLoS One. 2023 Jan 17;18(1):e0280140. doi: 10.1371/journal.pone.0280140 (PMC9844889; doi:10.1371/journal.pone.0280140)
Supplement: S6 Table — (DOCX) [file pone.0280140.s006.docx]

|  | **Univariate** | **Multivariate** | | | |
| --- | --- | --- | --- | --- | --- |
|  | *p-value* |  | Odds Ratio | CI | *p-value* |
| Mobility | ***<0.0001*** | No aid/Walking aid Wheelchair/Bedridden | 4.51  1.00 | 1.37-14.88 | ***0.01*** |
| Stone size | ***<0.0001*** | ≤5mm  6-10mm  ≥11mm | 9.97  1.79  1.00 | 2.19-45.46  0.61-5.29 | ***0.003***  *0.29* |
| History of stroke | ***0.047*** | yes  no | 7.39  1.00 | 1.43-38.22 | ***0.02*** |
| Age | ***<0.0001*** | <90 years  ≥90 years | 14.02  1.00 | 5.42-36.32 | ***<0.0001*** |
| Gender | ***0.002*** | male  female | 1.00  0.87 | 0.34-2.22 | *0.77* |
| Custodianship | ***<0.0001*** | yes  no | 0.2  1.00 | 0.04-1.14 | *0.07* |
| Stone location | ***0.001*** | proximal  distal | 1.00  1.39 | 0.53-3.66 | *0.51* |
| Diabetes mellitus | *0.63* |  |  |  |  |
| History of myocardial infarction | *0.25* |  |  |  |  |
| Indwelling urethral catheter | *0.59* |  |  |  |  |
| ASA -Scores | *0.07* |  |  |  |  |
| Anticoagulation | *0.86* |  |  |  |  |

Table 6: Logistic regression analyses : Likelihoods of receiving Dj-stent/PCN changes regularly instead of active stone treatment in elective settings.
