# Supplementary material for: Ureteral calculi in octogenarians and nonagenarians: Contemporary in-hospital management—A joint study by the endourological section of the Austrian Association of Urology
Source: PLoS One. 2023 Jan 17;18(1):e0280140. doi: 10.1371/journal.pone.0280140 (PMC9844889; doi:10.1371/journal.pone.0280140)
Supplement: S7 Table — (DOCX) [file pone.0280140.s007.docx]

|  | Octogenarians | Nonagenarians | *p-value* |
| --- | --- | --- | --- |
| n= | 643 (84.7%) | 116 (15.3%) |  |
| Stone location  proximal  Distal | 312 (50.4%)  307 (49.6%) | 80 (69.6%)  35 (30.4%) | *<0.0001* |
| Stone size (mm) | 7.3 (1-35) | 7.9mm (2-30) |  |
| Hospitalization (days) | 5.9 (1-50) | 5.5 (1-17) |  |
| ASA-Score  I  II  III  IV  V | 33 (6.2%)  224 (41.9%)  235 (44%)  40 (7.5%)  2 (0.4%) | 2 (2%)  17 (16.8%)  55 (54.5%)  27 (26.7%)  0 | *<0.0001* |
| Mobility  No aid needed  Walking aid  Wheelchair  Bedridden | 340 (57.3%)  172 (29%)  35 (5.9%)  46 (7.8%) | 30 (27.8%)  33 (30.6%)  14 (13%)  31 (28.7%) | *<0.0001* |
| Treatment acute setting  DJ-stent/nephrostomy replacement  Ureteroscopy  SWL in-situ | 206 (60.9%)  97 (24.7%)  35 (8.9%) | 39 (73.6%)  8 (15.1%)  6 (11.3%) | *0.11* |
| Treatment elective setting  DJ-stent/nephrostomy replacement  ureteroscopy  SWL in-situ | 23 (9.3%)  211 (85.7%)  12 (4.9%) | 33 (57.9%)  22 (38.6%)  2 (3.5%) | *<0.0001* |
| Stone free rates ureteroscopy | 79.8% (245/307) | 80% (24/30) | *0.98* |
| Intraoperative complications with URS (Clavien Dindo)  None  Ureteral injury/bleeding (I)  Perforation (IIIb – ureteral stent)  Avulsion (IIIb – open surgical repair)  Urosepsis (Grade IV – ICU unit)  Urosepsis (Grade V – death) | 278/308 (90.2%)  -  16/308 (5.2%)  2/308 (0.6%)  5/308 (1.6%)  1/308 (0.3%) | 28/30 (93.4%)  1/30 (3.3%)  -  -  1/30 (3.3%)  - | *0.58* |

Table 7: Treatment patterns in octogenarians vs. nonagenarians
